# The role of CD4^+^ T cells in visceral leishmaniasis; new and emerging roles for NKG7 and TGFβ

**DOI:** 10.3389/fcimb.2024.1414493

**Published:** 2024-05-31

**Authors:** Jinrui Na, Christian Engwerda

**Affiliations:** ^1^ QIMR Berghofer Medical Research Institute, Brisbane, QLD, Australia; ^2^ School of Medicine, University of Queensland, Brisbane, QLD, Australia

**Keywords:** visceral leishmaniasis, immunoregulation, CD4^+^ T cell, Nkg7 and TGFβ, IL-10

## Abstract

Visceral leishmaniasis is a potentially devastating neglected tropical disease caused by the protozoan parasites *Leishmania donovani* and *L. infantum* (*chagasi*). These parasites reside in tissue macrophages and survive by deploying a number of mechanisms aimed at subverting the host immune response. CD4^+^ T cells play an important role in controlling *Leishmania* parasites by providing help in the form of pro-inflammatory cytokines to activate microbiocidal pathways in infected macrophages. However, because these cytokines can also cause tissue damage if over-produced, regulatory immune responses develop, and the balance between pro-inflammatory and regulatory CD4^+^ T cells responses determines the outcomes of infection. Past studies have identified important roles for pro-inflammatory cytokines such as IFNγ and TNF, as well as regulatory co-inhibitory receptors and the potent anti-inflammatory cytokine IL-10. More recently, other immunoregulatory molecules have been identified that play important roles in CD4^+^ T cell responses during VL. In this review, we will discuss recent findings about two of these molecules; the NK cell granule protein Nkg7 and the anti-inflammatory cytokine TGFβ, and describe how they impact CD4^+^ T cell functions and immune responses during visceral leishmaniasis.

## Visceral leishmaniasis

Leishmaniasis is a significant neglected tropical disease caused by protozoan parasites of the genus *Leishmania* (Burza et al., 2018). The severity of *Leishmania* infections ranges from self-resolving skin sores known as cutaneous leishmaniasis (CL) to severe, systemic forms known as visceral leishmaniasis (VL) (Burza et al., 2018). VL is characterised by hepatosplenomegaly (simultaneous enlargement of the spleen and liver), pancytopenia (low levels of all blood cell types) and chronic inflammation in the bone marrow, lymph nodes and visceral organs. Typically, VL is fatal within two years if anti-parasitic chemotherapy is not administered, with the cause of death generally due to secondary infections, haemorrhages, and immunosuppression (Burza et al., 2018). In addition, as seen in the Indian sub-continent, some treated VL patients can develop Post-Kala-azar Dermal Leishmaniasis (PKDL), a disease characterised by skin rashes caused by parasites remaining in the skin without systemic parasite burden (Zijlstra, 2016). In East Africa, PKDL has a different pathogenesis and can develop concurrently with VL (Musa et al., 2002) Currently, 20 species of the genus *Leishmania* have been identified. Clinical manifestations of these pathogens in humans vary in severity due to interactions between parasite traits, vector biology, and host attributes (Colmenares et al., 2002).

The majority of *Leishmania* infections manifest as CL. Despite its self-resolving nature, CL can result in substantial social stigmatisation due to permanent scarring after the disease has resolved. Around 10% of CL cases progress to stronger manifestations that can result in severe disfigurements, such as mucocutaneous leishmaniasis, diffuse/disseminated CL, and leishmaniasis recidivans (Burza et al., 2018). Estimates by WHO point to 600,000 to 1 million cases of CL globally per year, while the total annual VL cases are estimated to be 50,000 to 90,000, approximately one-tenth that of CL. Despite its relatively low incidence, VL is the main contributor to deaths caused by *Leishmaniasis* due to its high lethality in untreated patients. VL is endemic in the Indian subcontinent, East Africa, and South America (Alvar et al., 2012). Two *Leishmania* species are responsible for most VL cases: *L. donovani* and *L. infantum*. The former is endemic in India and East Africa, while the latter is found in Central Asia, Central and South America, the Middle East, and the Mediterranean Basin (Alvar et al., 2012).

## Current status of disease control

Deaths from VL are predominantly found in underdeveloped regions, as poor infrastructure, malnutrition, and diseases, such as HIV, enhance the lethality of VL (Burza et al., 2018). Historically, India, Nepal, and Bangladesh accounted for over 50% of global VL cases. In 2005, these countries committed to eliminating VL as a health concern by 2015 (Burza et al., 2018). Elimination targets were set to one case per 10,000 people per year at the district or sub-district level, an incidence rate at which the disease is no longer a public health concern. Although the deadline has since been extended past 2015, significant progress has been made in eliminating VL, as the number of reported cases in these endemic countries declined by 96.2% from 2007 to 2021 (Organization, 2022).

Despite the substantial achievements in the effort against VL, the disease remains one of the top parasitic diseases regarding mortality and outbreak potential. Globally, an estimated 350 million people remain vulnerable to VL (Alvar et al., 2013). Factors behind the high outbreak potential include the conflict-driven migration of non-immune populations into endemic areas (Al-Salem et al., 2016), the emergence of VL in non-endemic areas by unknown means (Shrestha et al., 2019), and possible loss of herd immunity due to the declining number of cases in endemic regions (Rijal et al., 2019). In addition, the COVID-19 pandemic and subsequent reallocation of public health resources has significantly hampered efforts to control VL (Uwishema et al., 2022; Paul and Singh, 2023), and areas that have successfully reduced their incidence rates below the elimination target may see a resurgence of cases that lead to the return of their VL endemic status (Toor et al., 2020). Therefore, continued vigilance and effort are required to secure current gains and progress towards eliminating VL as a disease of public health concern.

## Treatment of visceral leishmaniasis

Traditional treatments for VL are based on pentavalent antimonial chemotherapies. Despite their effectiveness, these treatments also induced potent side effects and faced increasing drug resistance after decades of use (Sundar et al., 2000; Sundar and Chakravarty, 2010). Newer medicines, such as oral miltefosine and amphotericin B, exhibit lower toxicity but are also challenged by mounting drug resistance (Srivastava et al., 2011; Sundar et al., 2012; Alvar et al., 2013). Elimination of VL is complicated by asymptomatic patients who act as potential reservoirs of the parasite, and there are no approved effective vaccines available against human leishmaniasis (Dinc, 2022). These pressing issues highlight the need for novel approaches in VL treatments, such as immune-based therapies that enhance the host’s response against the parasite.

## Transmission cycle of Leishmania parasites

**Figure 1:**
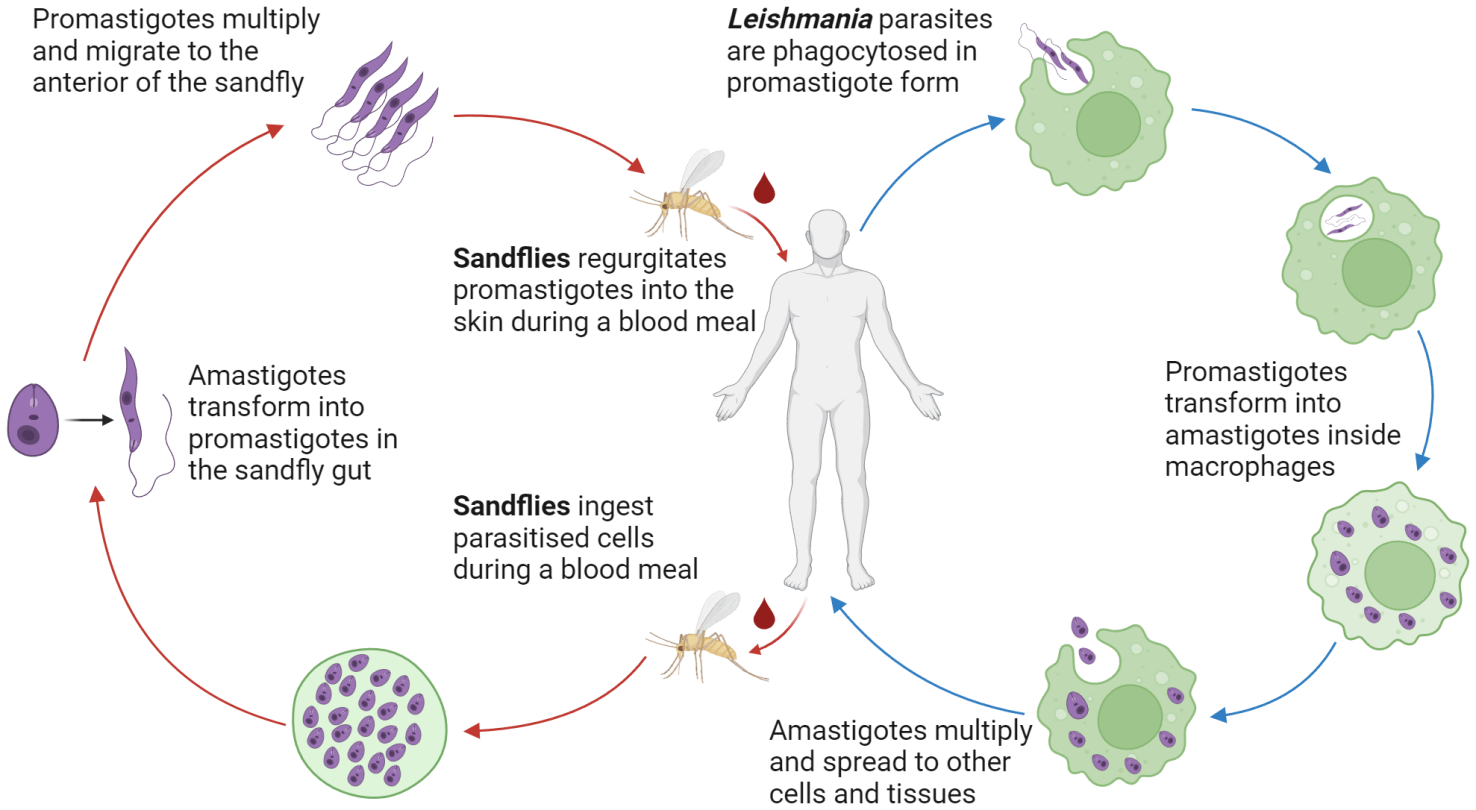
Lifecycle of Leishmania parasites. Dimorphic Leishmania parasites reside in the female Phlebotomine sand fly gut as flagellated extracellular promastigotes. Transformation to the immotile intracellular amastigote form occurs after phagocyte uptake of the parasite. Via numerous mechanisms, Leishmania parasites are able to resist lysis and replicate in phagocytes. Rupture of the host phagocyte leads to the continuation of the infection.

Transmission of *Leishmania* parasites occurs during the blood feeding of female *Phlebotomine* sand flies, in which the parasites in their motile, flagellated promastigote form are first regurgitated into the dermal layer of the host (Bates, 2007) (**Figure 1**). As the *Leishmania* species are obligate intracellular parasites, promastigotes must rapidly locate phagocytes, such as macrophages and neutrophils, to survive and replicate (Alexander et al., 1999). This is aided by the pro-inflammatory properties of the sandfly saliva, which acts as a chemo-attractant to phagocytes (Zer et al., 2001; Guimaraes et al., 2016). To further increase the efficiency of phagocytosis, the lipophosphoglycan (LPG) coat of *Leishmania* hijacks the body’s complement system to promote complement-mediated opsonisation while avoiding complement-mediated lysis of the parasite (Franco et al., 2012).

Once phagocytosed, *Leishmania* parasites transform into their round, immotile amastigote form. The LPG coat protects against reactive oxygen and nitrogen species (ROS and RNS) while establishing the parasite inside the phagolysosome (Franco et al., 2012). In addition, *Leishmania* parasites can delay the assembly of inducible nitric oxide synthetase (iNOS) on the phagolysosome (Pandey et al., 2016), decrease the rate of RNS production (Gaur et al., 2007), and disrupt the induction of iNOS and NOS through inhibition of oxidative stress (Olivier et al., 2012). These mechanisms create a favourable niche that enables the survival and replication of *Leishmania* parasites inside phagocytic cells.

### Murine models of experimental visceral leishmaniasis

Due to the impracticality of studying infected human organs, much of the current literature on VL is derived from experimental murine models. Unlike humans, common susceptible mouse strains such as C57BL/6 and BALB/c do not succumb to experimental visceral leishmaniasis (EVL) but instead develop an acute infection in the liver, followed by a life-long chronic infection in the spleen (Kumar and Nylen, 2012). This distinct pattern of immunity is not seen in human patients, as human VL manifests as a spectrum ranging from asymptomatic to severe disease characterised by multi-organ involvement (Faleiro et al., 2014). Parallels do, however, exist between the two, as asymptomatic VL patients exhibit effective immune responses akin to the mouse liver during infection. In contrast, symptomatic VL patients show impaired immune reactions similar to the mouse spleen (Faleiro et al., 2014). Studies on murine EVL can yield insights into immune networks that dictate parasite elimination versus persistence in human patients.

During murine EVL, liver infections are acute and self-resolving, with the reduction in parasite load occurring alongside granuloma formation. After the intravenous injection of *L. donovani*, resident macrophages (Kupffer cells in the liver - KCs) harbour the most parasites (McElrath et al., 1988; Honore et al., 1998). Due to their naturally impaired innate capacity to kill intracellular pathogens, the parasite load increases rapidly during the first weeks of infection (Wilson et al., 1996). Signalling between KCs drives their migration and fusion, resulting in the formation of the immunological complex known as granulomas, which consists of a core of fused KCs, an outer sheath of motile lymphocytes and a mix of other immune cells recruited by chemokine signalling from KCs (Murray, 2001).

Approximately one week after infection, recruited T cells accumulate in granulomas to become the predominant cell type (Stern et al., 1988). Although it was previously assumed that T cells were primed in the spleen and migrated to the liver to combat the infection, recent studies have shown that parasite-specific CD4^+^ T cells may be primed in other tissues and are sufficient to confer immunity (Engwerda and Kaye, 2000). Both CD4^+^ and CD8^+^ T cells are essential for granuloma formation (Stern et al., 1988). Still, whereas CD4^+^ T cells can be activated by both KCs and granuloma-associated dendritic cells (DCs) (Stanley and Engwerda, 2007), the activation of CD8^+^ T cells is restricted to KCs (Beattie et al., 2010).

Granulomas enable potent inflammatory cytokines produced by T helper 1 (Th1) cells, such as IFNγ and TNF, to be directed against concentrated pockets of parasitised KCs, which maximises their leishmanicidal capacity (Stanley and Engwerda, 2007). This results in effective clearance of the infection in the liver (Kaye et al., 2004). In addition, TNFα also plays a crucial role in the assembly and maturation of granulomas because, in its absence, granuloma formation is delayed, resulting in uncontrolled parasite growth in the first weeks of infection (Engwerda et al., 2004b). Granuloma maturation is attained by 2 to 4 weeks post-infection, and parasite burden declines rapidly up to the 8th week of infection (Murray, 2001). A sterile cure is not achieved in the liver; instead, a small population of parasites persist (Stanley and Engwerda, 2007). As the immune system will continue to mount a minor but sustained response against this small parasite population, its presence is thought to confer immunity to the host through constant antigen exposure, a process known as concomitant immunity (Stanley and Engwerda, 2007).

The progression of EVL in the mouse spleen occurs in a contrasting manner to the liver. Here granulomas do not form, instead parasites accumulate, and a chronic infection is established (Kaye et al., 2004). As a blood filter organ, the spleen removes foreign particles and pathogens from the blood (Mebius and Kraal, 2005). The splenic macrophages phagocytose around 95% of parasites introduced by intravenous injection (Gorak et al., 1998). Unlike KCs of the liver, these macrophages have a much stronger innate capacity to kill phagocytosed parasites, with around 50% of the initial inoculum being killed in the first 24 hours post-infection (Gorak et al., 1998).

Despite the initial anti-parasitic response in the murine spleen, chronic infection is eventually established by the third week after infection (Engwerda et al., 2004a). Multiple factors contribute to the failure of parasite control in the spleen, and the elevated levels of the anti-inflammatory cytokines IL-10 and TGFβ play a major role, as they result in the dampening of effector functions across a broad spectrum of immune cells such as macrophages, DCs and T cells (Nylen and Sacks, 2007). Additionally, as the infection progresses, the expression of exhaustion markers such as LAG-3, CTLA-4 and PD-1 on splenic T cells also increases (Jin et al., 2011), leading to the loss of effector functions such as the production of inflammatory cytokines and cytotoxic molecules (Edwards et al., 2023). This functional exhaustion in the spleen is most apparent in CD4^+^ T cells (Edwards et al., 2023).

A defining feature of VL in humans and mice is the enlargement of the spleen (splenomegaly) that occurs as the infection progresses to its chronic phase (Engwerda and Kaye, 2000). This change in spleen size and mass is accompanied by a loss of splenic microarchitecture, which includes the disruption of the marginal zone and white pulp, as well as the hypertrophy of the red pulp (Kaye et al., 2004). Interestingly, this loss of splenic microarchitecture is driven by TNF, the same inflammatory cytokine that plays a crucial role in the formation of granulomas and the subsequent clearance of parasite burden in the liver (Stanley and Engwerda, 2007). These disruptions lead to impaired cellular interactions, most notably between T cells and antigen-presenting cells (APCs), which further impairs the protective immune response in the spleen (Stanley and Engwerda, 2007).

### CD4^+^ T cell responses during visceral leishmaniasis

CD4^+^ T cells play critical roles in our body’s defence against pathogens (Tubo and Jenkins, 2014). They undergo specialisation after activation, yielding subsets that produce vital cytokines driving responses crucial for containing and eliminating specific infections. An essential facet of these responses is the emergence of specialised CD4^+^ T cells after pathogen control, initiating an anamnestic response upon reinfection (Kunzli and Masopust, 2023; Osum and Jenkins, 2023).

## T helper 1 cells

Th1 cells are a subset of CD4^+^ T cells characterised by the production of inflammatory cytokines such as IFNγ and TNF, driven by the master transcription factor T-bet (Szabo et al., 2003). For VL, generating an effective Th1 cell response is essential for controlling the infection (Reiner and Locksley, 1995; Murray and Delph-Etienne, 2000; Rosas et al., 2006; Singh and Sundar, 2018). APCs such as DCs are activated upon their phagocytosis of *Leishmania* antigens, and they subsequently process and present these antigens to naïve CD4^+^ T cells via MHC II. Signalling via the T cell receptor (TCR), along with co-stimulation via CD28, induces the production of IFNγ by T cells, and IFNγ in turn synergises with CD40 to stimulate IL-12 production by the APC, and also cooperates with TCR signalling to drive the first wave of T-bet expression (Schulz et al., 2009). Production and signalling of IL-12 by APCs results in the nuclear translocation of signal transducer and activation of transcription 4 (STAT4) in naïve CD4^+^ T cells. This results in a second wave of T-bet upregulation, as well as expression of the IL-12 receptor subunits, which then stabilises the Th1 cell fate after cessation of TCR signalling (Schulz et al., 2009). Continued signalling by IL-12 is also required to maintain IFNγ production by differentiated Th1 cells (Yap et al., 2000).

The production of pro-inflammatory cytokines such as IFNγ and TNFα by Th1 cells plays a key role in controlling VL infection. IFNγ is a potent macrophage activating factor, and its signalling activates naïve macrophages by driving their polarization into the inflammatory classically activated (M1) state (Tomiotto-Pellissier et al., 2018). The importance of IFNγ signalling in macrophages during VL is demonstrated by impaired parasite control in mice with macrophage-specific IFNγ signalling defects (Yap et al., 2000). TNF acts synergistically with IFNγ, and together, they increase the production of ROS and RNS, allowing parasitised macrophages to lyse the phagocytosed *Leishmania* amastigotes more effectively (Fromm et al., 2015).

As mentioned, an effective Th1 cell response is instrumental in parasite clearance in VL. Unsurprisingly, peripheral blood mononuclear cells (PBMCs) from symptomatic VL patients typically do not proliferate or produce limited IFNγ in response to *Leishmania* antigen (Sacks et al., 1987; White et al., 1992). Further, many symptomatic patients also present negative for leishmanin skin tests, indicating impaired cellular immune response against *Leishmania* antigens (Gidwani et al., 2009). Interestingly, PBMCs from cured patients regain their Th1 cell response to *Leishmania* antigens, only to revert to the symptomatic state upon co-culture with PBMCs collected before treatment from the same patient (Carvalho et al., 1989). These results indicate the presence of a cell-mediated immunosuppressive mechanism that regulates Th1 cell responses in symptomatic VL patients.

## T helper 2 cells

T helper 2 (Th2) cells are a subset of CD4^+^ T cells characterised primarily by the production of IL-4, as well as IL-5, IL-9 and IL13 (Walker and McKenzie, 2018). IL-4 signalling is key to the polarisation and maintenance of Th2 cells (Zhu, 2015). By activating STAT6 in naïve CD4^+^ T cells, IL-4 signalling drives the expression of the transcription factor GATA3, which synergises with STAT5 to promote IL-4 expression, forming a positive feedback loop that stabilises the Th2 programme (Yagi et al., 2011; Zhu, 2015). Th2 cells are mainly known to play an important role in promoting the healing of localised wounds caused by helminth infections (Chen et al., 2012), but in the context of *Leishmania* infections, Th2 cells generally promote parasite pathogenesis by suppressing inflammation (Sher and Coffman, 1992). It has been demonstrated in mouse models that IL-4-secreting CD4^+^ T cells exacerbated *Leishmania* infections (Scott et al., 1988). Similar results were also observed in humans, where VL patients who were nonresponsive to treatment had significantly higher levels of IL-4 production by T helper cells (Thakur et al., 2003). During VL, IL-4 and IL-13 inhibit Th1 cell activity and impair the production of reactive oxygen and nitrogen species in macrophages (Ho et al., 1992; Becker, 2004), while IL-5 and IL-9 also moderately promote VL progression (Uyttenhove et al., 1988; Nashed et al., 2000).

## T helper 17 cells

T helper 17 (Th17) cells are a subset of CD4^+^ T cells characterised primarily by the production of IL-17, IL-21 and IL-22 (Ouyang et al., 2008). The molecular basis of Th17 cell differentiation is complex, as different cytokine combinations can polarise naïve CD4^+^ T cells into Th17 cells with notable heterogeneity in the expression of immunoregulatory and proinflammatory genes (McGeachy et al., 2007; Ghoreschi et al., 2010; Lee et al., 2012). Th17 cells are defined as non-pathogenic or pathogenic based on their ability to induce tissue inflammation or autoimmunity (Schnell et al., 2023). Non-pathogenic Th17 cells can be polarised from naïve CD4^+^ T cells via the combination of IL-6 and transforming growth factor-beta (TGFβ), while pathogenic Th17 cells require IL-6 in combination with IL-1β and IL-23 or IL-6 in combination with TGFβ and IL-23 (Ghoreschi et al., 2010; Lee et al., 2012). The transcription factor RORγt is central to the Th17 cell programme (Ciofani et al., 2012). In addition, IL-6 and IL-23-induced STAT3 signalling is required for Th17 development (Yang et al., 2007), and STAT4 has been shown to support IL-17 production downstream of IL-12 (Mathur et al., 2007).

Th17 cells have mainly been recognised for their involvement in autoimmunity and control of fungal infections (Schnell et al., 2023), but their role in VL is less definitive due to contradictory results obtained from past studies. For example, levels of IL-17 and IL-22 were significantly increased following treatment of *L. donovani* infected mice, suggesting Th17 cytokines are associated with protection against VL infection (Khatonier et al., 2021). In addition, treatment-mediated protection against VL was able to be partially reversed in mice with the use of monoclonal antibodies against IL-17 and IL-23 (Ghosh et al., 2013). In human VL patients, Th17 cytokines were observed to be strongly associated with protection during a severe outbreak (Pitta et al., 2009). These results point to a protective role for Th17 cells during VL. Despite this, *Il17a* ^-/-^ mice were highly resistant to VL infection, as indicated by significant reductions in parasite levels compared to their wild-type (WT) counterparts, suggesting that IL-17 instead promotes susceptibility (Terrazas et al., 2016). These contradicting results could be due to the heterogeneity of Th17 cells caused by variations in the local cytokine environment of the diseased tissue.

## Regulatory T (Treg) cells

Anti-inflammatory cytokines such as IL-10 and TGFβ dampen the immune response by suppressing effector functions of T cells and APCs (Prud’homme, 2007; Ouyang et al., 2011). During VL, the production of anti-inflammatory cytokines determines the course of infection (Kima and Soong, 2013). A significant source of these anti-inflammatory cytokines is regulatory T (Treg) cells, which can be divided into two major subsets: natural (nTreg) and induced (iTreg). nTregs originate in the thymus, where thymocytes that show an intermediate level of self-reactivity develop expression of the transcription factor FOXP3 and subsequently emerge as a lineage distinct from conventional CD4^+^ T cells (Curotto de Lafaille and Lafaille, 2009). Under homeostatic conditions, nTregs are essential in keeping T cell responses controlled, as deficiencies in their number and function are associated with numerous autoimmune diseases (Long and Buckner, 2011). In contrast to nTregs, iTregs arise in the periphery from conventional CD4^+^ T cells in response to localised signals (Schmitt and Williams, 2013). Certain iTregs, such as the type 1 regulatory T (Tr1) cells, are essential in mediating the balance between parasite control and preventing inflammation-mediated tissue damage.

Past studies in human VL patients indicate an ineffective Th1 cell response occurs in patients unable to clear the parasite (Sacks et al., 1987; White et al., 1992). This failure is not due to defects in inflammatory cytokine production but rather due to the emergence of immunoregulatory mechanisms (Engwerda et al., 2014). IL-10 is often elevated in symptomatic VL patients and patients who develop PKDL after successful treatment for VL (Gasim et al., 1998). nTregs constitute a significant source of IL-10 during homeostasis. However, their contributions during VL are restricted to the preservation of splenic architecture and restriction of leukocyte expansion (Bunn et al., 2018). Instead, IL-10 from Tr1 cells is a significantly greater contributor to the suppression of the Th1 cell response during VL (Stager et al., 2006; Jankovic et al., 2007; Ansari et al., 2011; Schwarz et al., 2013; Bunn et al., 2018).

## Type 1 regulatory T cells

Tr1 cells are commonly defined as IL-10-producing Th1 cells (Chihara et al., 2016). Studies suggest the emergence of Tr1 cells, driven by IL-12 and IL-27 signalling (Awasthi et al., 2007; Apetoh et al., 2010; Neumann et al., 2014) and the transcription factors Blimp-1 (Neumann et al., 2014) and c-Maf (Apetoh et al., 2010), is likely a negative feedback mechanism that prevents excessive Th1-driven inflammation (Anderson et al., 2007; Sakaguchi et al., 2008). In Th1 cells, IL-12 promotes Blimp-1 expression via STAT4 (Neumann et al., 2014), while IL-27 drives IL-10 production through activation of STAT1 and STAT3 pathways (Stumhofer et al., 2007).

During VL, Tr1 cells promote pathogenesis by accumulating in the spleen and liver, and becoming the dominant source of IL-10 production, thereby dampening the effector functions of T cells and APCs, rendering the anti-parasitic immune response there ineffective (Nylen et al., 2007). Elevated levels of *BLIMP1* mRNA have been identified in CD4^+^ T cells of VL patients (Montes de Oca et al., 2016), and the induction of *BLIMP1* in CD4^+^ T cells is dependent on IL-27 and IL-12 signalling (Heinemann et al., 2014), both of which are elevated in the serum of VL patients (Khoshdel et al., 2009; Ansari et al., 2011). Further, Tr1 cells exert their immunomodulatory functions by producing anti-inflammatory cytokines IL-10 and TGFβ and expressing co-inhibitory receptors (CIRs) such as LAG3, PD-1 and CTLA4 (Song et al., 2021; Edwards et al., 2023).

**Figure 2:**
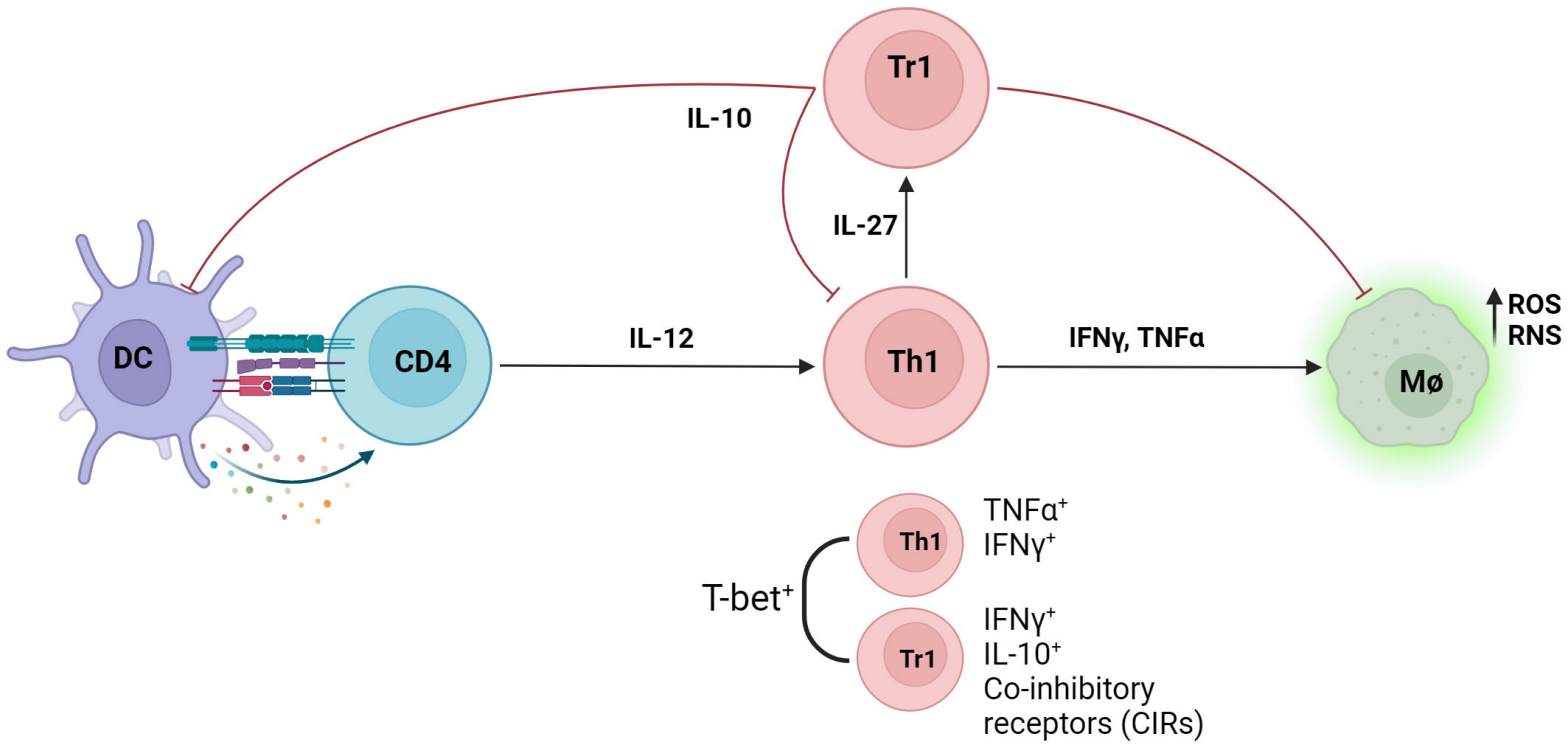
Infection with Leishmania parasites results in the generation of the anti-parasitic Th1 cell response. IL-12 produced by antigen presenting cells (APCs) induce the differentiation of naïve CD4^+^ T cells into inflammatory T helper 1 cells (Th1), defined as T-bet^+^ IFNγ^+^ CD4^+^ T cells. Inflammatory cytokines IFNγ and TNF produced by Th1 cells increases intracellular killing of phagocytosed parasites in macrophages by enhancing production of intracellular nitrogen and oxygen species (RNS/ROS). Type 1 regulatory (Tr1) cells, defined as IFNγ^+^ IL-10^+^, emerge from the Th1 cell population through IL-27 signalling and dampens the anti-parasitic response via IL-10. Both Th1 and Tr1 cells express T-bet, and co-inhibitory receptors (CIRs) are upregulated on Tr1 cells.

The immunoregulatory axis between inflammatory Th1 cells and regulatory Tr1 cells plays a key role during VL (**Figure 2**), as the balance of this axis is a key deciding factor between parasite control and persistence. Understanding the molecular mechanisms affecting this balance is a key step towards developing novel immune-based therapies against VL and other chronic inflammatory diseases where the Th1-Tr1 cell axis plays critical roles, such as cancer (Chaudhary and Elkord, 2016) and autoimmune diseases (Jeon et al., 2019).

### Emergence of natural killer granule protein 7 as a molecule of interest

Natural killer granule protein 7 (Nkg7) was discovered in 1993 as a 17 kDa type I integral membrane protein in human natural killer (NK) and T cells (Turman et al., 1993). The protein was found to be localised to the cytotoxic granule membranes of NK cells. During degranulation, the protein translocates from the granule membrane to the cell membrane, indicating a possible role in regulating the effector functions of several immune cell subsets (Medley et al., 1996). In the years after its discovery, the functional role of Nkg7 remained an underexplored topic, and detailed studies on *Nkg7* have only begun to emerge in recent years. Although little is known regarding its mechanistic function, current literature suggests that Nkg7 significantly promotes cytotoxic and inflammatory activity in multiple immune cell subsets.

Previously*, Nkg7* has been identified as part of the gene signature in Th1 and cytotoxic cells (Lund et al., 2005; Brouard et al., 2007; Jenner et al., 2009; Fu et al., 2015; Ayers et al., 2017), and the transcription factor T-bet had been found to bind to the promoter of Nkg7 (Jenner et al., 2009). More recently, Nkg7 was found to be upregulated in intratumor antigen-specific CD8^+^ T cells and NK cells, with *Nkg7*-deficient mice exhibiting impaired control of tumour growth (Li et al., 2022). Functionally, Nkg7 marks a subset of NK cell-derived extracellular vesicles enriched in granzyme B and Fas (Aarsund et al., 2022), and is also closely involved in numerous processes essential for the cytolytic activity of CD8^+^ T cells, such as cytolytic granule formation, trafficking, calcium release and synapse formation (Lelliott et al., 2022; Wen et al., 2022). Furthermore, Nkg7 has also been identified in the gene signatures of cytotoxic CD4^+^ T cells and their precursors (Patil et al., 2018), and *Nkg7*-expressing CD4^+^ T cells are enriched in vaccine-induced protective immune responses (Woodring et al., 2022).

**Figure 3:**
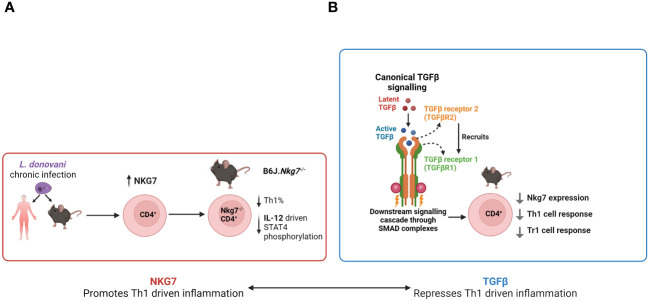
*The emerging roles of* Nkg7 and TGFβ in VL. **(A)** Following *Leishmania donovani* infection, CD4^+^ T cell *Nkg7* expression increases and promotes IL-12 signalling via STAT4 phosphorylation, thereby promoting Th1 cell development. **(B)** TGFβ signalling to CD4^+^ T cells inhibits *Nkg7* expression resulting in reduced Th1 and Tr1 cell development.

Recent work identified *Nkg7* as a gene of interest in VL. Differentially expressed genes (DEGs) in CD4^+^ T cells were identified in humans by comparing gene expression of peripheral CD4^+^ T cells in the same VL patients before and after drug treatment (Ng et al., 2020). Mouse splenic and liver CD4^+^ DEGs were obtained by comparing the naïve and infected states (day 56 post-infection). DEGs shared by both human and mouse splenic CD4^+^ T cells were defined as a core “inflammatory” signature imparted on CD4^+^ T cells during VL, and within this group, *Nkg7* was the most DEG (Ng et al., 2020). An important role for Nkg7 in the development of Th1 cells via IL-12-mediated STAT4 signalling was shown using Nkg7-deficinet mice (Ng et al., 2020) (**Figure 3A**).

Examination of *Nkg7* expression using a GFP reporter mouse revealed that while it is primarily expressed in NK and NKT cells in the naïve state, its expression by CD4^+^ T cells increased rapidly during experimental VL, peaking at day 28 post-infection (p.i.) in both spleen and liver (Ng et al., 2020), indicating a possible functional role of *Nkg7* in these cells. *In vitro* polarisation experiments conducted on naïve CD4^+^ T cells of the same reporter mice showed high levels of *Nkg7* expression in cells cultured under Th1 and Tr1 cell conditions, with the latter inducing the highest level of *Nkg7* expression out of all polarising conditions tested (Ng et al., 2020). Thus, NKG7 expression in this context suggests it is associated with the activation status of CD4^+^ T cells and not necessarily pro- or anti-inflammatory functions. Furthermore, *in vitro* titration experiments showed that *Nkg7* expression in CD4^+^ T cells increased in a dose-dependent manner with the addition of IL-27, while the reverse occurred with the addition of TGFβ (Ng et al., 2020). TGFβ is a known antagonist of *Blimp-1* expression (Neumann et al., 2014), an aforementioned key regulator of the development of Tr1 cells. These results suggest that Nkg7 may play an important role in the development and function of Tr1 cells (**Figure 3B**).

It was recently demonstrated that CD4^+^ T cells from IL-27 receptor-deficient mice (*IL27ra* ^-/-^) exhibit changes in metabolic activity during EVL (Montes de Oca et al., 2020). In particular, it was noted that *IL27ra* ^-/-^ CD4^+^ T cells have a greater frequency of cells with high mitochondrial mass but low mitochondrial potential, compared to wild-type (WT) CD4^+^ T cells. Given that *Nkg7* expression can be induced by IL-27 signalling, it is possible that, by extension, loss of *Nkg7* expression may mediate metabolic activity in CD4^+^ T cells. In support of this role, it was recently noted that Nkg7 is required for optimal acidic store-operated calcium entry (Wen et al., 2022), a process that proceeds degranulation in several cytolytic immune cell subsets.

### The regulatory role of transforming growth factor β on CD4^+^ T cells

TGFβ is a pleiotropic cytokine with numerous roles in the immune system (Sanjabi et al., 2017). TGFβ is initially translated as a ~50kDa precursor protein containing both TGFβ and the latency-associated peptide (LAP) (Robertson and Rifkin, 2016). Following dimerisation, this complex is associated with the latent TGFβ binding protein (LTBP), forming the large latent complex (LLC) (Miyazono et al., 1991). LAP is then excised in the trans-Golgi by furin-type enzymes while maintaining a non-covalent association with TGFβ, the complex of which is called small latent complex (SLC) (Hyytiainen et al., 2004). The LLC is then exported from the cell, where it binds to the extracellular matrix (ECM) via LTBP (Koli et al., 2005), becoming sequestered in the process. Alternatively, TGFβ could also be bound on the surface of Treg cells via GARP (glycoprotein A repetitions predominant), and this is thought to enhance TGFβ activation by concentrating it on the cell surface (Tran et al., 2009). Activation of latent TGFβ can occur physically (e.g. integrin-mediated) or chemically (e.g. protease-mediated) (Robertson and Rifkin, 2016).

TGFβ signalling is initiated by binding of the protein to the type II TGFβ receptor (TGFβRII), followed by the recruitment of type I TGFβ receptor (TGFβRI), which results in the formation of a heterodimer (**Figure 3**). Co-receptors, such as betaglycan, can modulate sensitivity to TGFβ isoforms (Cheifetz et al., 1990). Following the formation of a heterodimer complex, TGFβRII phosphorylates TGFβRI, and TGFβRI subsequently phosphorylates SMAD proteins (SMAD2/3) at two serine residues located near the C-terminal (Shi and Massague, 2003). The subsequent conformational change of the SMAD proteins allows them to bind to the common mediator SMAD4 via their MH2 domains, resulting in the formation of an SMAD complex (Kawabata et al., 1998). This then exposes a nuclear localisation signal and conceals a nuclear export signal, resulting in the nuclear accumulation of this complex and changes in gene expression (Feng and Derynck, 2005). Additionally, TGFβ is also able to signal in an SMAD-independent manner, such as the JNK, p38 and Erk signalling pathways (Zhang, 2009).

The immunosuppressive effect of TGFβ on the Th1 cell response has been demonstrated in both murine and human VL. Splenocytes and liver granuloma cells of the susceptible BALB/c strain produced reduced amounts of IFNγ in response to *Leishmania* antigen stimulation when compared to the genetically resistant C3H.HeJ strain (Wilson et al., 1998). This reduction was reversed when a neutralising TGFβ antibody was added to the culture, and levels of TGFβ were also found to be lower in the genetically resistant strain (Wilson et al., 1998). Further, TGFβ producing FOXP3^+^ Tregs accumulate at sites of infection, suggesting that TGFβ may play an immunosuppressive role in VL (Rodrigues et al., 2009). In humans, PBMCs of VL patients with drug-resistant parasites also produced significantly greater amounts of TGFβ in response to *L. donovani* antigen stimulation (Elmekki et al., 2016). Interestingly, *L. donovani* can exploit the suppressive functions of TGFβ to aid its survival (Barral-Netto et al., 1992; Barral et al., 1993), and it does so by expressing a cathepsin B-like cysteine protease to cleave the biologically active TGFβ from its latent complex (Somanna et al., 2002). Although numerous human and animal studies have highlighted the importance of TGFβ during VL, there remains a lack of knowledge detailing its effects on CD4^+^ T cell activity.

TGFβ is highly involved in the function and polarisation of CD4^+^ T cells. The primary effect of TGFβ signalling is akin to that of IL-10 in CD4^+^ T cells. T cell proliferation and macrophage activation, both of which are crucial to mounting an effective response against VL, are suppressed by the anti-inflammatory actions of TGFβ (Kehrl et al., 1986; Ding et al., 1990). Interestingly, it has been shown that TGFβ has a potent inhibitory effect on *Blimp1* expression in CD4^+^ T cells (Salehi et al., 2012; Neumann et al., 2014). As Blimp-1 is indispensable for the generation of Tr1 cells, this result suggests that TGFβ may also inhibit Tr1 cell formation. It is unclear the extent to which this may counteract the anti-inflammatory actions of TGFβ.

In addition, TGFβ can induce the expression of *FOXP3* in conventional CD4^+^ T cells, which in turn suppresses the activation and effector function of Th1 cells via the production of anti-inflammatory cytokines (Chen et al., 2003). However, it has been established that *Foxp3*-expressing Treg cells have limited involvement in suppressing anti-VL immunity (Bunn et al., 2018). Isoforms of TGFβ, such as TGFβ1 and TGFβ3, also play an important role in the generation of Th17 cells. Together with IL-6, TGFβ1 can polarise naïve CD4^+^ T cells into Th17 cells (Mangan et al., 2006). Further exposure to IL-23 induces the endogenous production of TGFβ3, which in turn acts as a potent driver of the pathogenic signature in Th17 cells (Lee et al., 2012). Given that Th1 cells dominate the adaptive immune response against VL, it is probable that the effect of TGFβ on Th17 differentiation is relatively inconsequential in this disease model.

The transcription factor TCF1 is highly expressed in T cells, playing an important regulatory role in both early and mature stages of T cells, although its effects vary between different T cell subsets (Zhao et al., 2022). In CD4^+^ T helper cells, TCF1 restrains Th1 and Th17 cell differentiation (Steinke and Xue, 2014), whilst promoting that of Th2 cells (Yu et al., 2009). In CD8^+^ T cells, TCF1 is indispensable for the generation and maintenance of a stem-like population responsible for replenishing exhausted effector cells during chronic infections (Utzschneider et al., 2016). Interestingly, ablation of TGFβ signalling results in reduced protein expression of TCF1 on CD8^+^ T cells in the lung during influenza (Wu et al., 2020), and in the spleen during LCMV infections (Hu et al., 2022). Studies examining the role of TCF1 in EVL are currently lacking. However, given its nature as a chronic infection, it is likely that the findings would reflect the results of other chronic models. The versatile actions of TCF1 and its link to TGFβ make it probable that altering TGFβ signalling will result in extensive changes to the biology of effector and memory T cells.

## Concluding remarks

TGFβ is a pleiotropic cytokine that is involved in multiple aspects of CD4^+^ T cell biology, such as the generation of regulatory T cells and the differentiation and function of effector subsets. Due to a lack of detailed functional studies on TGFβ during VL, it is unclear how these effects may interact and contribute to poor disease outcomes. Furthermore, TGFβ signalling is a potent antagonist to *Nkg7* expression on CD4^+^ T cells (**Figure 3**), but the nature of this interaction is unclear, and the biological implications of this interaction remain unknown. These gaps in our knowledge should be filled, as both TGFβ and Nkg7, and their associated cell signalling pathways, represent potential host-directed targets that could improve anti-parasitic immunity in response to either vaccination to drug treatment to prevent or treat VL.

## Author contributions

CE: Writing – original draft, Writing – review & editing. JN: Writing – original draft, Writing – review & editing.
